# The effect of hirudin on antagonisting thrombin induced apoptosis of human microvascular endothelial cells^1^

**DOI:** 10.1590/s0102-865020190010000006

**Published:** 2019-02-14

**Authors:** Jiangying Zhu, Xinyuan Pan, Bojie Lin, Guanyu Lin, Rohan Pradhan, Feiwen Long, Guoqian Yin

**Affiliations:** I MD, Department of Plastic and Aesthetic Surgery, The First Affiliated Hospital of Guangxi Medical University, China. Conception and design of the study, manuscript preparation, final approval.; II MD, Department of Plastic and Aesthetic Surgery, The First Affiliated Hospital of Guangxi Medical University, China. Acquisition, analysis and interpretation of data.; III IMM, Department of Plastic and Aesthetic Surgery, The First Affiliated Hospital of Guangxi Medical University, China. Technical procedures.; IV MM, Department of Ultrasonic, Maternal and Child Health Hospital of Guangxi Zhuang Autonous Region, China. Acquisition of data.; V MD, Department of Plastic and Aesthetic Surgery, The First Affiliated Hospital of Guangxi Medical University, China. Analysis and interpretation of data, critical revision.

**Keywords:** Hirudins, Thrombin, Apoptosis, Endothelial Cells

## Abstract

**Purpose:**

To investigate whether hirudin exerts its antithrombin action to decrease the ratio of Human Microvascular Endothelial Cells (HMVECs) apoptosis.

**Methods:**

Human microvascular endothelial cells (HMVECs) cultured in the third and fifth generations were used. HMVECs were divided into normal group, thrombin group (T group), natrual hirudin group (H group), thrombin + natrual hirudin group (T + H group), AG490 group, thrombin + AG490 group (T + AG490 group), natrual hirudin + AG490 group (H + AG490 group), thrombin + natural hirudin + AG490 (T + H + AG490 group).Apart from the normal group, the other groups were exposed to the relevant drugs for 24 hours.HMVEC apoptosis was assessed by flow cytometric and double Immunofluorescence of phosphorylation of JAK (P-JAK2) and TUNEL assay.

**Results:**

Compared with the normal group, in thrombin group the HMVECs apoptosis rate were significantly increased (P<0.05).The results indicated that the index of apoptosis and the apoptosis rate were improved in cultures treated by natural hirudin (T + H group), relative to cultures with thrombin only (T group). We found that the index of apoptosis and the apoptosis rate in the AG490 + thrombin group were higher than that in the hirudin + thrombin group (P<0.05). Double Immunofluorescence of p-JAK2 and TUNEL assays showed that cells were double positive for P-JAK2 uptake and TUNEL detection liquid binding.

**Conclusion:**

The natural hirudin and JAK2/STATs signal inhibitor AG490 could block the effects of thrombin. Natural hirudin could attenuate HMVECs apoptosis via antagonizing thrombin and it is suggested that this effect may occur by blocking the JAK2/STATs signaling pathway and this signaling pathways appears to be not the only pathway.

## Introduction

**Figure 1:**
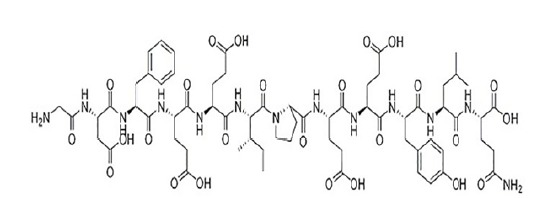
The chemical structure of natural hirudin.

Natural hirudin is a polypeptide isolated from the salivary glands of the leech, with a molecular weight of 7000 Dalton and composed of 64 to 66 amino acids. The tight structure formed by the disulfide bond at the N end of leech element can bind to the thrombin active site. The c-end is rich in acidic amino acid residues, and then binds to the fibrinogen binding site of thrombin, thus having a highly specific inhibition of thrombin and inhibiting the anticoagulation of thrombin (Fig. 1). 

Flap transplantation is widely used in plastic surgery, but it is plagued by necrosis of skin flaps after transplantation^1^. Postoperative anoxia and massive thrombin release can directly affect the survival of vascular endothelial cells, resulting in a decrease of blood vessels and a reduced blood supply after flap transplant operations. Thrombospondin-1(TSP-1) which is stimulated by thrombin^2^ ^,^ ^3^ is the first identified endogenous angiogenesis inhibitor^4^. It inhibits angiogenesis by inducing apoptosis in endothelial cells^5^. Our previous study found that natural hirudin treatment could decrease TSP-1 production, which may potentially contribute to the hirudin mediated effects of improving angiogenesis in ischemic flap tissue^6^. Hirudin is one of the most specific inhibitors of thrombin. It has obvious effects on the inhibition of platelet aggregation and direct thrombolytic and antithrombotic impact^7^. In our previous studies, Yin *et al.* ^8^, we determined the effect of natural hirudin on animal skin flap congestion and found that natural and recombinant hirudin could promote the expression of vascular endothelial growth factor, increase of Superoxide dismutase, decrease of Endothelin, Malonaldehyde^9^ etc. In addition, it was established that the mechanisms induced by hirudin might promote the angiogenesis, anticoagulation, antioxidation and antiinflammation reactions and the suppression of vasoconstrictions. 

Some studies have shown that JAK-2 appeared to be specifically TSP-1 upregulating^10^. However, there is little research on the signaling pathways in this regulation. 

It has been reported that JAK-STAT is one of the main pathways downstream of cytokine receptors and growth factor receptors by transducing signals from cell surface to the nucleus^11^. Cytokines or growth factors bind to the specific receptors, which could be recognized by the SH2 domains of STATs. Subsequently, STATs form homo- or hetero-dimers, and translocate to the nucleus where they bind to specific DNA elements and modulate the expression of target genes^11^. The JAK/STAT pathway plays a pivotal role in many important biological responses such as immune function, cell proliferation, differentiation and apoptosis. JAK2 is widely found in various tissues and cells. Further studies showed that PAR-1 activation-mediated tumor cell apoptosis is associated with tyrosine phosphorylation of JAK2 and STAT1, and translocation of STAT1 to the nucleus^12^. However, there are still no reports about the effect of hirudin in regulating the JAK2 pathway on thrombin induced apoptosis of microvascular endothelial cells in humans. In this report we hypothesize that hirudin, a specific thrombin inhibitor, might regulate the JAK2/STAT pathway on thrombin induced apoptosis of microvascular endothelial cells of human. We therefore investigated, whether natural hirudin could reduce the ratio of apoptosis induced by thrombin. AG490 is a tyrosine kinase inhibitor which plays various roles: It blocks JAKs kinase activation, which in turn blocks the transmission of its signal function to the nuclei. We thus explored whether AG490 could inhibit the JAK2/STATs pathway, and whether AG490 influenced the rate of apoptosis induced by thrombin. 

## Methods

###  Cell culture and reagents 

Commercial HMVECs was purchased from BeNa Culture Collection Co. Ltd. (Beijing, China). The cells were seeded in 89%H-DMEM medium with 10% fetal bovine serum (FBS, Gibco, Grand Island, NY, USA) and 1% Penicillin-Streptomycin Liquid (Beijing Solarbio Science & Technology Co., Ltd) at 37ºC in a 5% CO_2_ incubator. HMVECs cell cultures in the third and fifth generations were used. The cultured HMVECs were divided into a normal group, a thrombin group (2U/ml thrombin, T group), a natural hirudin group (2ATU/ml natural hirudin, H group), a thrombin and hirudin group (2U/ml thrombin + 2ATU/ml hirudin, T + H group), an AG490 group (50μmol/L AG490), a thrombin + AG490 group (2U/ml thrombin + 50 μmol/L AG490, T + AG490 group ), a hirudin + AG490 group (2ATU/ml hirudin + 50 μmol/L AG490, H + AG490 group), thrombin and hirudin and AG490 group (2U/ml thrombin+2ATU/ml hirudin+50μmol/L AG490, T + H + AG490). Apart from the normal group, the other groups were exposed to the relevant drugs for 24 hours, and then the corresponding indicators were detected.

Lyophilized natural hirudin powder (Patent number ZL03113566.8, Lot number KK-001) was provided by Nanning JinXueHuang Bioengineering Co. Ltd. (Guangxi, China). Thrombin was purchased from Sigma-Aldrich (USA) and AG-490 was purchased from Target Molecule Crop (USA). The phosphorylation-JAK2 (ab68268) antibodies were purchased from Abcam. The goat anti-rabbit second-fluorescence were also purchased from Abcam.

###  Flow cytometric detection of HMVEC apoptosis 

Cell apoptosis was quantified using the Annexin V: FITC apoptosis detection kit I (BD Biosciences Pharmingen, US) and analyzed by flow cytometry. Annexin V was considered as a sensitive index of early stage apoptotic cells, while PI was the indicator of advanced stage apoptotic cells. Briefly, the cells were re-suspended in 500 µl binding buffers and stained consecutively by 5 µl Annexin V-FITC and 5 µl PI. We then analyzed HMVECs by flow cytometry (FACSCalibur; BD Biosciences, Franklin Lakes, NJ, USA) to differentiate apoptotic cells from necrotic cells. Both HMVECs stained Annexin+/PI- and Annexin+/PI+ in the flow cytometric analyses were considered as apoptotic cells. 

###  Double immunofluorescence 

To examine differentiation from apoptosis cells into expressed P-JAK2 cells, double immunofluorescence staining for TUNEL and P-JAK2 was performed according to manufacturer’s instructions (Roche Molecular Systems, Pleasanton, CA, USA). To immuno-stain, cells were fixed with 4% paraformaldehyde for 45 min, subsequent immersion in PBS with 0.1% Triton x-100 on the ice for 2min, blocked with goat serum for 15 min, probed with primary antibody(P-JAK2) overnight at 4°C, and labeled with secondary antibodies (Goatantirabbit IgG) conjugated to TUNEL detection liquid and DAPI. The immunoreactions were observed under confocal MS (LSM510 META NLO, Carl Zeiss). The TUNEL-positive cells that showed green nuclear staining and all of the cells with blue nuclear DAPI staining were counted within five randomly chosen fields under a high-power magnification. The index of apoptosis was expressed as the ratio of positively stained apoptotic myocytes/the total number of myocytes counted ×100%.

###  Statistical analysis 

All data were expressed as mean ± SD. The statistical significance of the deviation between the groups was determined by Compare Student’s T-test or One-Way ANOVA. Statistical analyses were performed using SPSS version 13.0, and a value of p < 0.05 was considered statistically significant.

## Results

**Figure 2:**
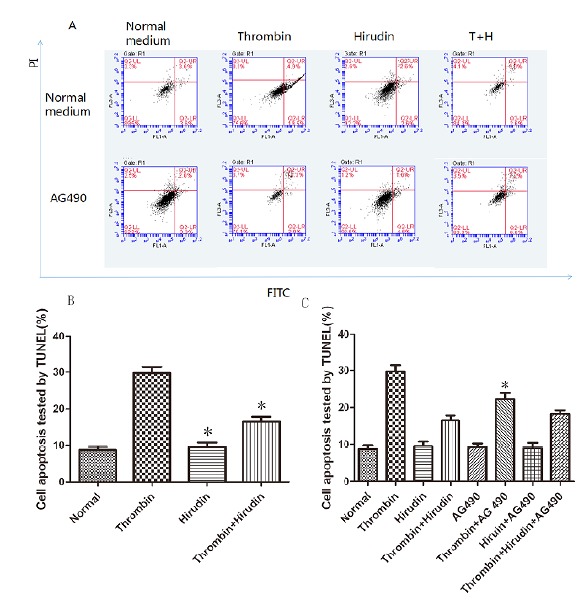
Hirudin protection against thrombin-induced apoptosis in HMVECs. HMVECs were stimulated with thrombin, treated with hirudin or treated with hirudin together with AG490 for 24h following the culture development for 24h. (**A**) HMVECs were analyzed by flow cytometry. The results are expressed as the cell ratio after staining with propidium iodide and deoxyuridine triphosphate-fluorescein. (**B**) The results revealed that hirudin treatment lead to a reduction in apoptosis when compared with thrombin stimulation only. * indicates the P <0.05 *vs.* the thrombin group, n=5 for each group. The apoptosis rate in Hirudin group was lower than that in thrombin group, which was equivalent to that of normal group. * marks the P <0.05 *vs.* thrombin group, n=5 for each group. (**C**) AG490 protected against Thrombin- induced apoptosis in HMVECs. The results revealed that AG490 treatment lead to a reduction in apoptosis when compared with thrombin stimulation only. * represents the P <0.05 *vs.* the thrombin group, n=5 for each group.

The HMVEC apoptosis rate of each group was detected by flow-cytometric analysis, after 24 hours of drug treatment. The evaluation of the results indicated no significant difference of cell apoptosis rate between normal group and natural hirudin group (P>0.05, Fig. 2 A,B). Thus, natural hirudin concentration of 2ATU/ml was considered not to be cytotoxic to HMVECs. However, the cell apoptosis rate in the thrombin group was 4-fold higher than that in the normal group (P <0.05, Fig. 2 A,B). In the T+H group, the cell apoptosis rate was decreased compared with the thrombin group (P <0.05, Fig. 2 A,B), but it was larger than the normal group (P <0.05, Fig. 2 A,B). To investigate whether the hirudin could attenuate HMVEC apoptosis via antagonizing thrombin by blocking the JAK2 signaling pathway, 50μmol/L of AG490 was added 30min before thrombin treatment. There was no significant difference of cell apoptosis rate between the normal group and the AG490 group (P>0.05, Fig. 2 A,C). This indicates that AG490 has no toxic effects on cells. The treatment of cells with AG490 produced a significant inhibition of apoptosis (P <0.05, Fig. 2 A,C), which was even larger than that of the T+H group (P <0.05, Fig. 2 A,C). These results suggest the possibility that hirudin could attenuate HMVEC apoptosis via antagonizing thrombin, not only by blocking the JAK2 signaling pathway, but also by other signaling pathways.

**Figure 3:**
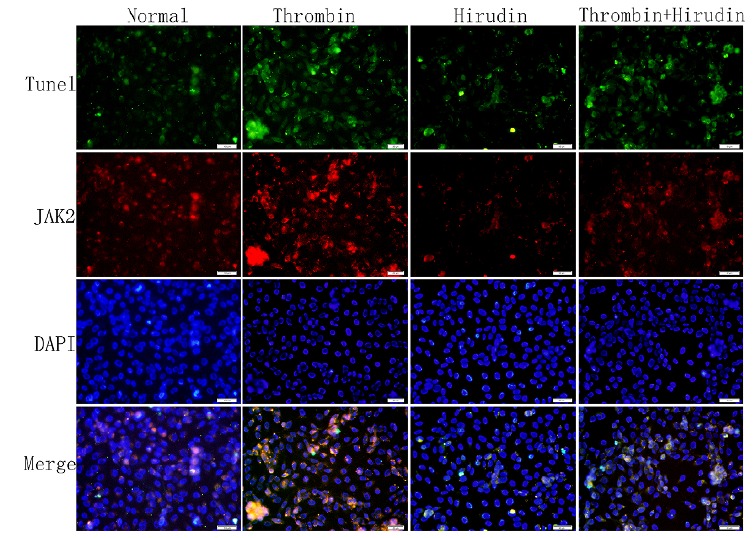
The index of apoptosis and the expression of P-JAK2. Double immunofluorescence staining for TUNEL and for p-JAK2 was performed to determine whether P-JAK2 was expressed in apoptotic cells. HMVECs were analyzed by TUNEL and P-JAK2 double immunofluorescence staining. The respective images (magnification, x200) as well as the statistical results of TUNEL staining, have verified these pathways. The results were calculated by determining the ratio of DNA damaged cells that were stained green and red. The representative images showed that P-JAK2 was expressed in apoptotic cells.

**Figure 4:**
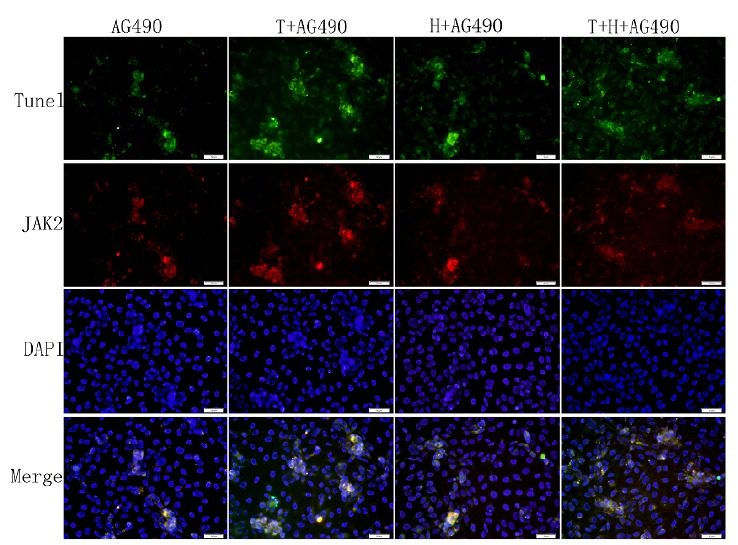
The effects of AG490 on apoptosis index and expression of JAK2.

**Figure 5:**
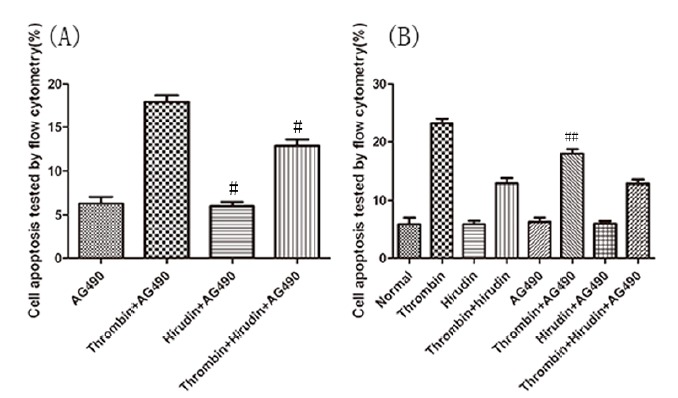
The Statistical analysis chart of the apoptosis index. (**A**) The results revealed that hirudin treatment lead to a reduction in apoptosis and P-JAK2 expression when compared with thrombin stimulation only. #marks the P <0.05 *vs.* the thrombin group, n=5 for each group. (**B**) The results showed that AG490 treatment lead to a reduction in apoptosis and P-JAK2 expression when compared with thrombin stimulation only. ## represents the P <0.05 *vs.* the thrombin group, n=5 for each group. The results are expressed as the cell index of apoptosis.

To determine whether P-JAK2 was expressed in apoptotic cells, double immunofluorescence staining for TUNEL and p-JAK2 was performed. We observed that the cells were double positive for P-JAK2 uptake and TUNEL detection liquid binding (Figs. 3 and 4). The normal group and hirudin group exhibited a similar apoptosis index (P>0.05, Figs. 3 and 5A). In the thrombin group, the expression of P-JAK2 and the apoptosis index was increased, compared with the normal group (P <0.05, Figs. 3 and 5A). In the T+H group, the expression of P-JAK2 and the cell apoptosis index were decreased compared with the thrombin group (P <0.05, Figs. 3 and 5A). However, the apoptotic rate in group T+H was lower than that in group T, but was distinctly larger than that of the normal group (P <0.05, Figs. 3 and 5A). There was no significant difference of the expression of P-JAK2 and the cell apoptosis index between the normal group and the AG490 group (P>0.05, Figs. 4 and 5B). The expression of P-JAK2 and the cell apoptosis index were decreased in the T+H group and the T+AG490 group (P <0.05, Figs. 4 and 5B), while that of the T+AG490 group was elevated compared with the T+H group (P <0.05, Figs. 4 and 5B). This result is consistent with the findings of our above experiments. As demonstrated in Figs. 2-5, hirudin abolished the stimulating effect of thrombin on the P-JAK2 expression. Taken together, these data suggest that hirudin may suppress the expression of P-JAK2 by suppressing the thrombin signaling. 

## Discussion

Random pattern skin flaps are used to repair skin defects during plastic and reconstructive surgery, however, flap necrosis remains a challenging problem. Our previous studies have shown that natural hirudin can increase flap viability in animal experiments^8^. Flap necrosis can be induced by many conditions such as ischemia , hypoxia, activation of the coagulation system, vascular thrombosis, venous congestion and inflammation^13^. The individual causes can’t be studied separately in vivo experiments. The work presented here aimed to study the molecular mechanism that mediates the anti-apoptosis effect of natural hirudin in human endothelial cells by in-vitro experiments. The results show that natural hirudin does protect HMVECs and decrease cell apoptosis via antagonizing thrombin, by blocking the JAK2 signaling pathway.

Thrombin is the key product of the coagulation system and is produced following injury. The thrombin generation level is associated with ischemia. Evidence indicates that a large amount of thrombin is produced immediately after brain ischemia, and high concentrations of thrombin within ischemic brain tissue is likely to cause more brain injury^14^ ^,^ ^15^. Nagy *et al.* ^16^ have found that thrombin may cause brain microvascular endothelial cell injury after adding thrombin to a culture. Moreover, high concentrations of thrombin could result in apoptosis of endothelial cells^17^. Other authors have found that the apoptosis of vascular endothelial cells can damage the integrity of vascular endothelium and increase vascular permeability^18^. The increase of vascular permeability is also caused by thrombin which is a direct consequence of an injury of vascular endothelial cells. Our results in this study ultimately show, that thrombin significantly increased the human microvascular endothelial cells apoptosis. 

The JAK-STAT signaling pathway can be induced by a number of cytokines^19^ (such as Interleukin family) and cell growth factors^20^ (such as epidermal growth factor and fibrogenic growth factor), all of which can play a specific and effective biological function in cell proliferation, differentiation or apoptosis, immune function regulation and tumor formation^21^. The cytokines bind to receptors on the cell membrane, and the activated receptors produce JAK binding sites, which are activated with phosphorylation. Further studies showed that PAR-1 activation-mediated tumor cell apoptosis is associated with tyrosine phosphorylation of JAK2 and STAT1, and translocation of STAT1 to the nucleus^12^.

PAR-1 has been considered to be the most important thrombin receptor and it plays a major role in thrombin signal transduction^22^. AG490 blocks JAKs kinase activation, which in turn blocks the transmission of its signal function to the nuclei. In the present study, we found that thrombin significantly increased the human microvascular endothelial cells apoptosis, while AG490 can block this effect.

Hirudin is a highly specific and potent inhibitor of thrombin; it can bind to thrombin and thereby inhibit thrombin signaling. Our earlier study showed that the circulation in over-dimensioned random pattern skin flaps could be significantly improved by hirudin^23^. In addition, topical application of natural hirudin could reduce the local inflammatory response, alleviating capillary permeability and improving blood circulation and angiogenesis, thus resulting in an improved survival rate of ischemic flaps^8^. We also found that a cross-talk from p38 MAPK to ERK pathway appears to exist in ischemic flap tissue, and hirudin may exerts its angiogenesis effect via inhibiting the thrombin-induced negative cross-talk of p38 MAPK-ERK^6^. However, the molecular pathways that regulate the process of hirudin-induced anti-vascular endothelial cell apoptosis in vitro experiments remain unclear. In this study, we could show that thrombin significantly increased the human microvascular endothelial cells apoptosis, while natural hirudin could reduce this effect of thrombin. The AG490 also could reduce the effect of thrombin, but the cell apoptosis rate was higher than that of the hirudin treatment. We interpret this to mean that hirudin could attenuate HMVEC apoptosis by antagonizing thrombin, not only by blocking the JAK2 signaling pathway, but also by other signaling pathways.

## Conclusions

The natural hirudin can inhibit the apoptosis of vascular endothelial cells by blocking antithrombin and blocking the JAK2 pathway. The JAK2 pathway is a significant, but not the only signal pathway that plays a critical role in regulation of apoptosis after natural hirudin treatment: our results of the experiments with added AG490 indicate the presence of other signaling pathways. This study provides an experimental basis and elucidates some of the active pathways for the clinical application of natural hirudin to prevent the flap ischemia in humans.
